# The management of bronchus intermedius complications after lung transplantation: A retrospective study

**DOI:** 10.1186/1749-8090-7-8

**Published:** 2012-01-20

**Authors:** Shahrzad M Lari, Francois Gonin, Arlette Colchen

**Affiliations:** 1 Lung disease and Tuberculosis Research Center, School of Medicine, Mashhad University of Medical Sciences, Mashhad, Iran; 2 Interventional bronchoscopy unit, Thoracic Surgery and lung transplantation department, Foch hospital, Suresnes, France

## Abstract

**Background:**

Airway complications following lung transplantation remain a significant cause of morbidity and mortality. The management of bronchial complications in Bronchus Intermedius (BI) is challenging due to the location of right upper bronchus. The aim of this study was to analyze the results of BI Montgomery T-tube stent in a consecutive patients with lung transplantations.

**Methods:**

Between January 2007 and December 2010, 132 lung transplantations were performed at Foch Hospital, Suresnes, France. All the patients who had BI Montgomery T-tube after lung transplantation were included in this retrospective study. The demographic and interventional data and also complications were recorded.

**Results:**

Out of 132 lung transplant recipients, 12 patients (9 male and 3 female) were entered into this study. The indications for lung transplantation were: cystic fibrosis 8 (67%), emphysema 3 (25%), and idiopathic pulmonary fibrosis 1 (8%). Most of the patients (83%) had bilateral lung transplantation. The mean interval between lung transplantation and interventional bronchoscopy was 11.5 ± 9.8 (SD) months. There was bronchial stenosis at the level of BI in 7 patients (58.3%). The Montgomery T-tube number 10 was used in 9 patients (75%). There was statistically significant difference in Forced Expiratory Volume in one second (FEV1) before and after stent placement (p = 0.01). The most common complication after stent placement was migration (33%).

**Conclusion:**

BI complications after lung transplantation are still a significant problem. Stenosis or malacia following lung transplantation could be well managed with modified Montgomery T-tube.

## Background

Airway complications are still a potential cause of morbidity and mortality after lung transplantation even with the considerable improvements in anastomotic techniques and immunosuppressive drugs [1,2]. The reported incidence of airway complications is about 7 to 18% with a mortality rate of 2 to 4% [3].

Since systemic arterial blood flow is not preserved during engraftment, lung transplantation is completely different from other transplantations [4]. Revascularization of the bronchial arterial flow may take up to 4 weeks [3,5]. Thus the viability of donor's bronchus is completely dependent to the retrograde collaterals from low pressure pulmonary circulation [3,6]. During this period, the anastomotic site is prone to ischemia and may develop varying degrees of necrosis and dehiscence [7]. Therefore airway complications have been mainly attributed to ischemia of donor bronchus during the immediate post transplant period [3,8]. Other attributing factors like primary graft dysfunction [9], pre and post pulmonary infection [3,10], rejection [4], intense immunosupression [4], and prolonged mechanical ventilation [11] are associated with increased risk of anastomotic airway complications.

The clinical presentation of airway complications varies from focal infection, necrosis, dehiscence, and granulation tissue formation to stenosis and malacia [7].

**Figure 1:**
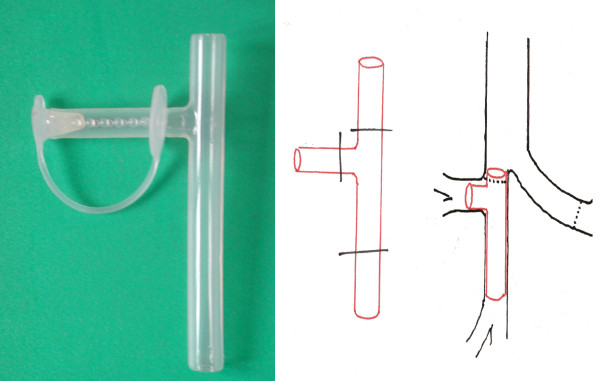
**Montgomery T-tube placement in Bronchus Intermedius (BI) position**.

The Bronchus Intermedius (BI) is especially prone to ischemia after lung transplantation [7]. Different therapeutic modalities, like endobronchial dilatation, laser, cryosurgery, and endobronchial stents, are used for the treatment of BI stenosis depending on the severity of stenosis. The main problem with stent placement in BI is the possible obstruction of right upper lobe orifice [7] or restenosis at the upper part of the stent. In Foch Hospital, the modified Montgomery T-tube (Novatech, France) as shown in Figure 1 was applied as stent for BI stenosis or malacia. In this technique, after measuring, cutting, and manual smoothening the distal ends, the tracheotomy arm of the T tube is placed in the upper lobe bronchus to maintain its patency.

The purpose of this study was to analyze the results of the modified Montgomery T-tube in BI of lung transplant patients over the past 4 years.

## Materials and methods

We reviewed the data of all the lung transplantations which were performed in the Lung Transplantation and Thoracic Surgery department of Foch Hospital, Suresnes, France between January 2007 and December 2010. All the patients with Montgomery T-Tube in BI location were entered into this retrospective study.

Heart lung, bilateral lung, and single lung transplantation were performed according to the standard methods [2,12]. End-to-end anastomosis with no revascularization techniques had been applied.

All of the patients received triple immunosuppressive therapy consisting of cyclosporine, azathioprine or mycophenolate mofetil, and prednisone. Also chemoprophylaxis against pneumocystis jiroveci, and cytomegalovirus were given to the patients.

Surveillance bronchoscopy and transbronchial lung biopsy were performed immediately after transplantation and subsequently on postoperative day 7, month 1, 2, 3, 6, 9, 12, 18, and 24.

The severity of bronchial ischemia at the level of anastomosis was assessed as following:

0: No ischemia.

1: Mild ischemia (one isolated ischemic patch or one at the level of anastomosis)

2: Moderate ischemia (circumferential anastomotic ischemia)

3: Severe ischemia (circumferential anastomotic ischemia and ischemia in segmental bronchi with or without dehiscence)

**Figure 2:**
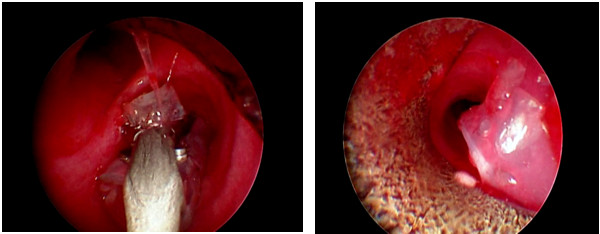
**Introduction of T-tube in right main bronchus**.

**Figure 3:**
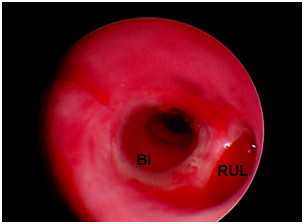
**T-tube in bronchus intermedius (BI) and right upper lobe (RUL)**.

In the case of airway complications including stenosis, malacia, or obstructive necrotic tissues, rigid bronchoscopy (KARL STORZ, Germany) was applied. It was performed under general anesthesia and jet ventilation. Depending on the pathological finding, the suitable interventions including mechanical dilatation, laser, and cryotherapy were performed. The dilatation was used when there was a significant decline in forced expiratory volume in one second (FEV1) with no other etiology or narrowing of the airway > 50% of predicted diameter. Additionally, the Montgomery T-tube with appropriate size was inserted when there was significant stenosis or malacia (Figure 2, 3) but not necessarily after the first dilatation.

Spirometry was performed within 1 month intervals and also before and after any bronchial interventions when it was possible.

### Statistical analysis

The data were analyzed using the Statistical Package for Social Sciences (Version 14.0; SPSS

Inc., Chicago, IL). The variables are presented as percentages and means ± standard deviations. Descriptive statistics were used to summarize the demographic characteristics of the patients. For comparison of the variables T-test was applied. *P *values less than 0.05 were considered significant.

## Results

Out of 132 lung transplantations, 12 patients (male/female: 9/3) with mean age of 37 ± 13.89 years were enrolled into our study. The indications for lung transplantation were: cystic fibrosis 8 (67%), emphysema 3 (25%), and idiopathic pulmonary fibrosis 1 (8%). Most of the patients (10/12) had bilateral lung transplantation. The frequency of severe bronchial ischemia in one month after transplantation was 41.5% in comparison of 8% in 7 days after operation (p = 0.03). The main clinical data of the patients are shown in Table 1.

**Table 1:** The demographic and transplantation parameters of the patients.

Clinical and interventional parameters	Values
**Age**	37 ± 13.89*

**Etiology**	Cystic fibrosis:8 (67%) Emphysema:3 (25%) Idiopathic Pulmonary Fibrosis:1 (8%)

**Type of transplantation**	Bilateral: 9 (75%) Bilateral+Kidney: 1 (8%) Single lung (right): 2 (17%)

**Ischemic time (hours)**	Right: 4.7 ± 0.98* Left: 3.9 ± 2.28*

**Bronchial ischemia (7^th ^day)**	Grade 0:0 Grade 1: 2 (17%) Grade 2: 9 (75%) Grade 3:1(8%)

**Bronchial ischemia (1^st ^month)**	Grade 0:0 Grade 1: 2 (17%) Grade 2: 5 (41.5%) Grade 3:5(41.5%)

*The data are shown as mean ± SD.

There was bronchial stenosis at the level of BI in 7 patients (58.3%). The Montgomery T-tube was placed in all of the patients. Out of 12 patients, 3 (25%) had the size of 12 and others (75%) size 10. The left side stent was placed in 3 (25%) patients.

**Figure 4:**
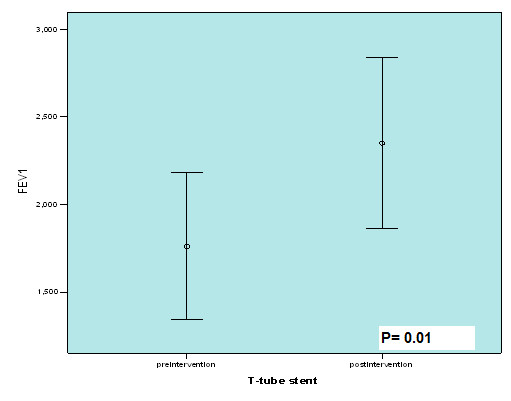
**The comparison of FEV1 pre and post Montgomery T-tube stent**.

The data about rigid bronchoscopy are shown in Table 2. The mean total times of 7.4 ± 2.6 rigid bronchoscopy were performed in patients. There was statistically significant difference between the mean FEV1 before and after the stent placement (1762 ± 586.70 and 2351 ± 681.34 respectively, p = 0.01) Figure 4.

**Table 2:** The data of interventional bronchoscopy in patients.

**Variable**	Value
**Indication**	Stenosis:8 (66%) Malacia: 2 (17%) Stenosis+ Malacia: 2 (17%)

**Location of stenosis**	Anastomosis: 4 (33%) Bronchus Intermedius (BI): 7(59%) Anastomosis + BI: 1(8%)

**Interval between transplantation and intervention (months)**	11.5 ± 9.8*

**Duration of stent replacement (months)**	6 ± 6.6*

**Complications**	Migration: 4(33%) Stenosis: 3(25%) Obstruction:2(17%) Infection: 1(8%) No complications:2(17%)

**FEV1 (ml)**	Before stent replacement:1762 ± 586.70* After stent replacement:2351 ± 681.34*

*The data are shown as mean ± SD.

The minimum duration of follow up was 6 months. The most common complication after stent placement was migration of the stent (33%). The Montgomery T- tube was extracted in 9 patients (75%). Seven patients (58.3%) needed a new stent.

## Discussion

Despite the progressions of surgical methods in lung transplantation during recent years, airway complications are still a major concern in this field. The airway complications after lung transplantation can be divided in two phase: the early phase (during the first month after transplantation) including anastomotic infection, necrosis, or dehiscence which is mainly due to bronchial ischemia and the late phase including excess granulation tissue, bronchomalacia, and airway stenosis [3,7].

As we mentioned earlier, the main mechanism of bronchial ischemia is the interruption of bronchial blood flow after anastomosis [6,13]. Bronchial stenosis is the most common airway complication and often occurs in patients who have experienced anastomotic necrosis, dehiscence, or infection [3,14]. In our study, overall 10 patients (84%) had significant stenosis.

During recent decades, many surgical methods have been proposed for preventing of bronchial ischemia [3,15], but only end-to-end anastomosis is the recommended technique for it [3]. In our study, all of the lung transplantation was performed with end-to-end anastomosis method.

Since bronchial ischemia is a main underlying factor in occurrence of airway complications, assessment of the severity of ischemia seems to be necessary. We designed a classification staging according to the severity of ischemia on bronchoscopy. In our study 10 patients (83%) had moderate to severe bronchial ischemia one month after transplantation. The comparison of bronchial ischemia in day 7 and month 1 after transplantation showed the progressive nature of ischemia.

It is well documented that most airway complications after lung transplantation occur at the anastomotic site but sometimes nonanastomotic bronchial stenosis may also occur [16]. Although the exact pathogenesis of this type of stenosis is not clear, but alloreactive injury, ischemic damage, and infection may concern [16]. Several cases of bronchial stenosis distal to the anastomotic site in the BI have been reported [17]. In our study, 59% of bronchial stenosis was in BI without involvement of anastomosis. This type of stenosis is known as the vanishing bronchus intermedius syndrome that may be detected as late as 12 months post transplant [7,17]. The mean interval between transplantation and stent placement was 11.5 months in this study.

Management of the bronchial stenosis depends on severity of narrowing. Different modalities like mechanical dilatation, laser, cryotherapy, and stent are applied in appropriate indications [18]. In the case of nonanastomotic bronchial stenosis, the therapeutic options are not different. But it is necessary to keep a stent in the correct position without occlusion of the right upper lobe bronchus. We used modified Montgomery T-tube as shown in Figure 1, 2, and 3. By this method, the right upper bronchus will be kept open.

In our study, the most common complication was migration. If we could use an appropriate diameter T-tube stent which is fit to the BI, the complication may be lowered. The special designed T-tube stents suitable for BI in diameter may lower the risk of complications like migration.

## Conclusions

Airway complications after lung transplantation are still a managing problem; especially when they are located in BI. For maintaining the patency of BI, especial stents are required because of the position of right upper bronchus. We used modified Montgomery T-tube for this purpose. But due to complications like migration, especially designed stents compatible with diameter of BI and main bronchus are nowadays applied. So we expect that the efficiency of this endoscopic procedure will be increased because there is no real efficient treatment of this type of post lung transplantation bronchial complication.

## List of abbreviations

BI: Bronchus Intermedius; FEV1: Forced Expiratory Volume in one second

## Competing interests

The authors declare that they have no competing interests.

## Authors' contributions

AC and FG participated in performing the procedure of stent placement and management of the patients. FG also participated in design of the study. SML participated in design of the study, statistical analysis, and draft the manuscript. All the authors read and approved the manuscript.
